# Sustained Virological Response After Direct-Acting Antiviral Therapy in Hepatitis C Virus-Infected Individuals With and Without Decompensated Liver Cirrhosis: A One-Year Follow-Up Study

**DOI:** 10.7759/cureus.79766

**Published:** 2025-02-27

**Authors:** Shailja, Shruti Radera, Sumit Rungta, Amar Jeet, Amita Jain

**Affiliations:** 1 Microbiology, King George's Medical University, Lucknow, IND; 2 Gastroenterology, King George's Medical University, Lucknow, IND; 3 Clinical Microbiology, King George's Medical University, Lucknow, IND

**Keywords:** compensated liver cirrhosis disease, decompensated liver cirrhosis disease, direct acting antivirals, hepatitis c virus, sustained virological response

## Abstract

Introduction

Hepatitis C is a significant global health concern, causing many deaths. In the National Viral Hepatitis Control Program (NVHCP), direct-acting antiviral (DAA) therapy (sofosbuvir and velpatasvir, without ribavirin) is used to achieve sustained virological response (SVR) in hepatitis C virus (HCV)-infected individuals. The duration is longer for decompensated liver cirrhosis disease (DCLD) patients (24 weeks) compared to compensated liver cirrhosis disease (CLD) patients (12 weeks). The present study was planned to assess SVR in patients with HCV infection, both with CLD and DCLD, at 12 weeks, 6 months, and 1 year after treatment initiation.

Methods

This pilot study enrolled 100 treatment-naïve chronic hepatitis C patients, with 50 having CLD and 50 having DCLD. Serum samples were collected from these patients before treatment initiation, and follow-up samples were collected at 12 weeks, 6 months, and 1 year after initiation of treatment to monitor SVR in both groups by real-time polymerase chain reaction (PCR).

Results

Among 50 DCLD patients, three died within the first three months of treatment. The remaining 47 achieved an undetectable viral load at 12 weeks (100%). By six months, two more patients had died, and one experienced viral relapse, resulting in a 97.9% SVR. However, by one year, all surviving patients had no detectable viral load. The DCLD group had a 10% mortality rate (5/50), including three deaths within 12 weeks (one from variceal bleeding and two from non-liver-related causes), and two post-treatment despite achieving SVR. Mortality was not linked to viral load, suggesting that liver function and disease severity play a more significant role in patient outcomes. All CLD patients achieved SVR at 12 weeks after initiation of therapy (100%), which persisted at six months and one-year follow-up.

Conclusion

DAA therapy is highly effective, achieving a 100% SVR rate and sustained liver function improvement in HCV-infected patients with liver disease.

## Introduction

Globally, chronic hepatitis C virus (HCV) infection remains a significant public health challenge, affecting an estimated 58 million people, with approximately 1.5 million new infections annually. While HCV prevalence varies across regions, low- and middle-income countries, including India, bear a substantial portion of the disease burden [1]. In India, HCV prevalence is estimated to range from 0.09% to 2.02%, with regional variations influenced by healthcare access, screening programs, and risk factors, such as unsafe medical practices and blood transfusions [2,3]. Given this considerable burden, effective antiviral therapies are crucial to achieving national and global HCV elimination targets.

The introduction of highly effective direct-acting antiviral (DAA) agents has revolutionized the treatment of HCV infection, achieving high sustained virologic response (SVR) rates. These medications, with their favorable safety profiles, have demonstrated significant benefits. They reduce Model for End-stage Liver Disease (MELD) scores, improve quality of life, and decrease morbidity associated with cirrhosis [4,5]. Compared to pegylated interferon alpha and ribavirin-based therapies, DAAs offer significantly higher cure rates, shorter treatment durations, and excellent tolerability [6]. In India, the availability of generic DAAs has significantly lowered costs, improving accessibility. Under the National Viral Hepatitis Control Program (NVHCP), DAAs are provided free of cost for uncomplicated cases at the district level and complicated cases at specialized centers (MTCs, or medical treatment centers), further enhancing treatment access [7]. However, challenges remain in ensuring widespread coverage, particularly in rural areas, due to disparities in healthcare infrastructure, awareness, and affordability.

SVR is defined as undetectable or unquantifiable HCV RNA 12 weeks or 24 weeks after the completion of treatment. This is considered indicative of a cure for chronic HCV infection [8]. Successful HCV treatment has been demonstrated to reduce the risk of hepatic failure, hepatocellular carcinoma (HCC), liver-related deaths, and all-cause mortality. Within one year of achieving SVR, approximately one-quarter of patients with compensated cirrhosis no longer exhibit liver stiffness in the cirrhotic range [9].

The NVHCP of India recommends treating patients with HCV infection using DAAs. For patients with decompensated liver cirrhosis disease (DCLD), the NVHCP advises treatment with sofosbuvir and velpatasvir with ribavirin for 12 weeks, or sofosbuvir and velpatasvir without ribavirin for 24 weeks. For patients with compensated liver cirrhosis disease (CLD), a 12-week treatment with sofosbuvir and velpatasvir is recommended [7].

The present study was planned to monitor the SVR at 12 weeks, 6 months, and 1 year after sofosbuvir/velpatasvir treatment in treatment-naïve chronic hepatitis C patients with CLD and DCLD. The secondary objectives include assessing mortality rates, virological relapse, and changes in liver function parameters over the follow-up period.

## Materials and methods

This pilot study was conducted in the Postgraduate Department of Microbiology and the Department of Gastroenterology at King George’s Medical University, Lucknow, India, a tertiary care referral and teaching center. The Ethics Committee of King George's Medical University issued approval for this study (approval no. 1948/Ethics/2023). The study duration was extended to 18 months, running from January 22, 2023, to July 20, 2024.

The study enrolled 100 treatment-naïve patients aged 18 years or older with HCV infection, including DCLD (50) and CLD (50) patients. According to the NVHCP, DCLD was defined as Child-Turcotte-Pugh (CTP) Class B or C, with clinical signs and symptoms of liver disease decompensation, including ascites, jaundice, hepatic encephalopathy, variceal bleeding, or hepatorenal syndrome [7]. These patients were treated with sofosbuvir (400 mg) and velpatasvir (100 mg) for 24 weeks. CLD was characterized by CTP Class A and the absence of esophageal or gastric varices observed during upper gastrointestinal endoscopy, without a history of variceal bleeding, and/or liver stiffness measurement ≥12.5 kPa on transient elastography, supported by evidence from abdominal ultrasound [7]. These patients received sofosbuvir (400 mg) and velpatasvir (100 mg) for 12 weeks.

Exclusions comprised individuals with previous interferon or DAA therapy, hepatitis B virus (HBV) or human immunodeficiency virus (HIV) coinfection, chronic kidney disease, severe cardiac or pulmonary conditions, a history of organ transplantation, malignancy, pregnancy, or unwillingness to participate.

Serum samples were collected, and viral load was estimated by in-house quantitative real-time polymerase chain reaction (PCR) at the time of initiation of treatment and after 12 weeks, 6 months, and 1 year of initiation of treatment to monitor SVR. Clinical assessments, including medical history, liver function tests, abdominal ultrasonography, esophago-gastro-duodenoscopy, and FibroScan findings, were recorded from patient case sheets. During follow-up, adherence was monitored through patient self-reports and medical records, with missed dose inquiries and prescription refill reviews ensuring treatment compliance.

The statistical analysis was done using Epi Info 7 Statistical Analysis Software (Centers for Disease Control and Prevention (CDC), Atlanta, GA, USA). The values were represented as number (%) and mean ± SD. A paired t-test was used for all comparisons.

## Results

The mean age and range of the total study population were 50.47 ± 12.15 and 22-88 years, respectively. The age range for DCLD was 22-88 years, and the age range for CLD was 23-70 years.

The frequency of chronic hepatitis C was equally distributed between females and males, with a female-to-male ratio of 1:1 (50:50) in the study population. Among patients with DCLD, the female-to-male ratio was 24:26, while, for patients with CLD, the ratio was 26:24.

The mean viral load at therapy initiation was 2.6 × 10^6^ ± 5.6 × 10^6^ IU/mL for DCLD patients and 4.9 × 10^6^ ± 1.2 × 10^7^ IU/mL for CLD patients. Mean FibroScan values were 34.95 ± 15.95 kPa for the DCLD group and 22.05 ± 9.01 kPa for the CLD group. In the DCLD group, 82.0% of cases were in Class B, and 18.0% were in Class C, with a mean score of 7.92 ± 1.34. All CLD patients were in Class A, with a mean score of 5.12 ± 0.32. In the DCLD group, 66% of cases had small, and 34% of cases had large esophageal varices.

Table 1 shows that, in DCLD patients at the 12-week follow-up, three patients died, while the remaining 47 cases were negative for hepatitis C virus-ribonucleic acid (HCV-RNA). At the six-month follow-up, one patient from the remaining 47 cases became positive for HCV-RNA, was excluded from further follow-up, and was put on a different therapy. Two patients died during the one-year follow-up, and the remaining 44 remained HCV-RNA negative. All DCLD patients showed significant changes in their CTP scores from baseline to follow-up. There were statistically significant improvements in all baseline parameters after 12 weeks, 6 months, and 1 year after initiation of treatment (Table 1).

**Table 1:** Changes in laboratory parameters during and after DAAs therapy in HCV infected patient with decompensated liver cirrhosis disease Statistical test: A paired t-test was used for all comparisons, and p-values indicate statistical significance compared to baseline. *One patient who turned HCV-RNA positive was excluded from further follow-up. PT, Prothrombin time; INR, International normalized ratio; SGOT, Serum glutamic oxaloacetic transaminase; SGPT, Serum glutamate pyruvate transaminase; S. albumin, Serum albumin; S. bilirubin, Serum bilirubin; S. ALP, Serum alkaline phosphatase; CTP, Child-Turcotte-Pugh; DAAs, Direct-acting antivirals; HCV-RNA, Hepatitis C virus ribonucleic acid

Laboratory parameter	Baseline (n = 50)	12 weeks (n = 47)	6 months (n = 47)	1 year (n = 44)	p-value (vs. baseline)
HCV-RNA negative	0	47	46*	44	-
Hemoglobin (g/dL)	9.97 ± 1.6	10.7 ± 1.3	11.4 ± 1.11	12.27 ± 0.77	<0.001 (12w, 6m, 1y)
PT (seconds)	19.22 ± 4.06	12.04 ± 0.83	12.11 ± 0.47	12.36 ± 0.69	<0.001 (12w, 6m, 1y)
INR	1.46 ± 0.38	1.01 ± 0.04	1.00 ± 0.009	1.01 ± 0.01	<0.001 (12w, 6m, 1yr)
S. bilirubin (μmol/L)	1.19 ± 0.59	0.77 ± 0.24	0.63 ± 0.20	0.49 ± 0.10	<0.001 (12w, 6m, 1yr)
SGOT (U/L)	99.9 ± 63	50.45 ± 19.02	37.8 ± 3.3	37.55 ± 3.54	<0.001 (12w, 6m, 1yr)
SGPT (U/L)	70.08 ± 56	46.7 ± 18	37.2 ± 8.56	41.59 ± 22.1	<0.001 (12w, 6m, 1yr)
S. albumin (g/dL)	3.33 ± 0.63	4.16 ± 0.46	4.2 ± 0.49	4.09 ± 0.27	<0.001 (12w, 6m, 1yr)
S. ALP (U/L)	281 ± 125	192 ± 71	162.9 ± 55	155.38 ± 33.9	0.001 (12w, 6m, 1yr)
CTP score (%)					
Class A (5-6)	0%	100%	100%	100%	<0.001 (12w, 6m, 1yr)
Class B (7-9)	82%	0%	0%	0%	<0.001 (12w, 6m, 1yr)
Class C (10-15)	18%	0%	0%	0%	<0.001 (12w, 6m, 1yr)

Table 2 shows that all CLD patients achieved SVR at 12 weeks of treatment and remained HCV-RNA free throughout the follow-up period (one year). In the CLD group, no significant changes were observed in baseline prothrombin time (PT), international normalized ratio (INR), serum bilirubin, and CTP scores. All patients were in CTP Class A throughout the study period. Hemoglobin changes were not significant. Its inclusion remains clinically relevant as an indicator of health, treatment tolerance, and anemia risk. Significant changes were noted in serum glutamic oxaloacetic transaminase (SGOT), serum glutamate pyruvate transaminase (SGPT), and serum alkaline phosphatase levels at 12 weeks and beyond, and in serum albumin at six months and one-year follow-up (Table 2).

**Table 2:** Changes in laboratory parameters during and after DAAs therapy in HCV infected patient with compensated liver cirrhosis disease Statistical test: A paired t-test was used for all comparisons, and p-values indicate statistical significance compared to baseline. PT, Prothrombin time; INR, International normalized ratio; SGOT, Serum glutamic oxaloacetic transaminase; SGPT, Serum glutamate pyruvate transaminase; S. albumin, Serum albumin; S. bilirubin, Serum bilirubin; S. ALP, Serum alkaline phosphatase; CTP, Child-Turcotte-Pugh; DAAs, Direct-acting antivirals; HCV-RNA, Hepatitis C virus ribonucleic acid

Laboratory parameter	Baseline (n = 50)	12 weeks (n = 50)	6 months (n = 50)	1 year (n = 50)	p-value (vs. baseline)
HCV-RNA negative	0	50	50	50	-
Hemoglobin (g/dL)	12.65 ± 1.67	12.09 ± 1.10	11.83 ± 1.01	12.19 ± 0.75	0.090 (12w); 0.392 (6m); 0.058 (1yr)
PT (seconds)	14.14 ± 1.12	12.4 ± 1.18	12.40 ± 1.19	12.0 ± 0.17	1.000 (12w, 6m, 1yr)
INR	1.105 ± 0.13	1.06 ± 0.6	1.13 ± 0.10	1.013 ± 0.017	1.000 (12w, 6m, 1yr)
S. bilirubin (μmol/L)	0.82 ± 0.39	0.71 ± 0.23	0.62 ± 0.20	0.50 ± 0.15	1.000 (12w, 6m, 1yr)
SGOT (U/L)	110 ± 59	34.4 ± 12.8	31.7 ± 7.6	29.88 ± 5.6	<0.001 (12w, 6m, 1yr)
SGPT (U/L)	114.09 ± 82	36.07 ± 14	33.84 ± 6.6	31.2 ± 5.51	<0.001 (12w, 6m, 1yr)
S. ALP (U/L)	300 ± 128	170 ± 42	169.4 ± 63.7	148.5 ± 37	<0.001 (12w, 6m, 1yr)
S. albumin (g/dL)	4.10 ± 0.44	4.6 ± 0.65	4.7 ± 0.51	5.39 ± 0.62	0.564 (12w); 0.034 (6m); <0.001 (1yr)
CTP class	A	A	A	A	-

## Discussion

This study demonstrates a 100% SVR rate at 12 weeks among DCLD patients treated with sofosbuvir and velpatasvir without ribavirin. However, at the six-month follow-up, one patient tested positive for HCV-RNA, which subsequently reverted to negative following retreatment. In patients with CLD, a consistent 100% SVR was achieved at 12 weeks, 6 months, and the 1-year follow-up, highlighting the efficacy of the treatment regimen across different stages of liver disease.

The study suggests sofosbuvir and velpatasvir as a first-line treatment for HCV infection in DCLD patients for 12/24 weeks without ribavirin, showing 100% effectiveness. Our results are supported by other studies recommending sofosbuvir and velpatasvir without ribavirin for the treatment of HCV infection. For instance, Zhang et al. reported that patients treated with sofosbuvir and velpatasvir without ribavirin for 12 weeks achieved an SVR rate of 91.5%, comparable to SVR rates with sofosbuvir and velpatasvir plus ribavirin [10]. Similarly, Mangia et al. reported an SVR rate of 98.4% [11], while Kumar et al. demonstrated an SVR rate of 100% at 12 weeks, aligning with the findings of this study [2]. Conversely, some studies, like An et al. in Korea, reported an 87% SVR after 12 weeks, lower than the present study's findings [12]. Krassenburg et al. reported a 91% SVR at 12 weeks of treatment [13]. These varying SVR rates could be due to differences in patient populations, treatment regimens, and treatment duration.

The present study demonstrates a significant reduction in the CTP score among patients with DCLD after 12 weeks of treatment, with the score decreasing from 7.92 ± 1.34 to 5.25 ± 0.44 (p < 0.001). This improvement continued, with the CTP score further reducing to 5.06 ± 0.24 at six months (p < 0.001) and to 5.0 ± 0 at the one-year follow-up (p < 0.001). Based on the CTP score, all patients initially classified as Class B or Class C transitioned to Class A following treatment, indicating a marked improvement in liver function and disease severity. The findings from our study align well with other studies conducted in the past in India and Egypt [2,14,15].

DAA therapy in DCLD patients leads to improved liver function, demonstrated by a significant reduction in cellular injury post-treatment of chronic HCV infection. Consistency across multiple studies strengthens the validity of the findings and suggests that the observed improvement in CTP scores in patients with DCLD is a robust outcome of the treatment [14,16,17].

Both the DCLD and CLD patients experienced statistically significant decreases in SGOT, SGPT, serum alkaline phosphatase, and INR levels, along with increased serum albumin at 12 weeks, 24 weeks, and 6 months post-therapy follow-up, indicating improvement in liver function tests. HCV-related cirrhosis treated with DAAs is known to show improvement in liver function tests, as reported by other investigators [15,18].

In the DCLD group, a 10% mortality rate was observed, with three deaths occurring within the first 12 weeks of treatment initiation and two within one year. These deaths were primarily attributed to advanced liver disease and complications such as hepatic decompensation and variceal bleeding, rather than treatment-related adverse effects. Notably, no correlation was found between mortality and viral load, suggesting that liver function, disease severity, and overall treatment response play a more significant role. Kumar et al. reported 7% mortality in similar patients [2]. Despite achieving SVR, patients with pre-existing portal hypertension still face risks of variceal progression, liver decompensation, and mortality [14,19].

One patient experienced viral relapse at the six-month follow-up. While the exact cause remains uncertain, potential factors, such as NS5A resistance-associated variants (RAVs), may have contributed. Despite initially achieving an undetectable viral load after treatment, the patient later had detectable HCV-RNA, suggesting the possible presence of residual viral variants below the detection limit at the end of therapy. These variants may have subsequently replicated, leading to relapse [20]. This highlights the need for ongoing monitoring and follow-up care to promptly detect and manage potential relapses.

The positive outcomes suggest that successful treatment with DAAs not only clears the HCV, but also leads to beneficial changes in liver health. Several studies have demonstrated improvements in the severity of liver disease following SVR [5,10,13,15,21].

These findings highlight the importance of monitoring and managing potential complications during treatment and addressing unrelated factors impacting patient outcomes.

Limitations

This study has several limitations, including a small sample size, short follow-up duration, and the absence of randomization and blinding - all of which may reduce statistical power and impact generalizability. The lack of HCV genotype data and RAV testing limits insights into treatment response variability. Additionally, fibrosis regression and HCC risk post-SVR were not assessed, and potential biases in study design could further affect the findings. Future research should focus on larger cohorts, extended follow-ups, and improved study design to enhance validity and broader applicability.

## Conclusions

DAA therapy is highly effective, with an excellent SVR rate of 100% in HCV-infected patients with DCLD and CLD. Improvement in liver function was observed after treatment. These improvements were sustained, emphasizing the long-term benefits of successful DAA therapy.

## References

[1] (2025). Hepatitis C. https://www.who.int/news-room/fact-sheets/detail/hepatitis-c#:~:text=For%20World%20Hepatitis%20Day%202023,the%202030%20hepatitis%20elimination%20target.

[2] Kumar A, Kumar S, Deep A (2019). Efficacy of directly acting antiviral drugs for HCV in real life: experience from a tertiary care centre of North India. J Evid Based Med Healthc.

[3] Puri P, Anand AC, Saraswat VA (2014). Consensus statement of HCV task force of the Indian National Association for Study of the Liver (INASL). Part I: Status report of HCV infection in India. J Clin Exp Hepatol.

[4] Curry MP, O'Leary JG, Bzowej N (2015). Sofosbuvir and velpatasvir for HCV in patients with decompensated cirrhosis. N Engl J Med.

[5] Charlton M, Everson GT, Flamm SL (2015). Ledipasvir and sofosbuvir plus ribavirin for treatment of HCV infection in patients with advanced liver disease. Gastroenterology.

[6] Gupta V, Kumar A, Sharma P, Arora A (2017). Newer direct-acting antivirals for hepatitis C virus infection: perspectives for India. Indian J Med Res.

[7] (2025). Diagnosis and management of viral hepatitis national guidelines for 2018. https://nvhcp.mohfw.gov.in/Guidelines.

[8] Simmons B, Saleem J, Hill A, Riley RD, Cooke GS (2016). Risk of late relapse or reinfection with hepatitis C virus after achieving a sustained virological response: a systematic review and meta-analysis. Clin Infect Dis.

[9] Singh S, Facciorusso A, Loomba R, Falck-Ytter YT (2018). Magnitude and kinetics of decrease in liver stiffness after antiviral therapy in patients with chronic hepatitis C: a systematic review and meta-analysis. Clin Gastroenterol Hepatol.

[10] Zhang W, Zhang J, Tang S (2023). Efficacy and safety of sofosbuvir-based regimens in hepatitis C patients with decompensated cirrhosis: a systematic review and meta-analysis. J Clin Transl Hepatol.

[11] Mangia A, Cenderello G, Copetti M (2019). SVR12 higher than 97% in GT3 cirrhotic patients with evidence of portal hypertension treated with SOF/VEL without ribavirin: a nation-wide cohort study. Cells.

[12] An J, Park DA, Ko MJ, Ahn SB, Yoo JJ, Jun DW, Yim SY (2022). Direct-acting antivirals for HCV treatment in decompensated liver cirrhosis patients: a systematic review and meta-analysis. J Pers Med.

[13] Krassenburg LA, Maan R, Ramji A (2021). Clinical outcomes following DAA therapy in patients with HCV-related cirrhosis depend on disease severity. J Hepatol.

[14] Bakr D, Hassan I, Abd-Elmaksood E (2021). Outcomes of direct acting antiviral therapy for treatment of HCV infection in patients with decompensated liver cirrhosis. Med J Viral Hepat.

[15] Garg G, Dixit VK, Shukla SK (2018). Impact of direct acting antiviral drugs in treatment naïve HCV cirrhosis on fibrosis and severity of liver disease: a real life experience from a tertiary care center of North India. J Clin Exp Hepatol.

[16] Belli LS, Duvoux C, Berenguer M (2017). ELITA consensus statements on the use of DAAs in liver transplant candidates and recipients. J Hepatol.

[17] Romano J, Sims OT, Richman J (2018). Resolution of ascites and hepatic encephalopathy and absence of variceal bleeding in decompensated hepatitis C virus cirrhosis patients. JGH Open.

[18] Verna EC, Morelli G, Terrault NA (2020). DAA therapy and long-term hepatic function in advanced/decompensated cirrhosis: real-world experience from HCV-TARGET cohort. J Hepatol.

[19] Libânio D, Marinho RT (2017). Impact of hepatitis C oral therapy in portal hypertension. World J Gastroenterol.

[20] Ahmed A, Felmlee DJ (2015). Mechanisms of hepatitis C viral resistance to direct acting antivirals. Viruses.

[21] Zeng H, Li L, Hou Z, Zhang Y, Tang Z, Liu S (2020). Direct-acting antiviral in the treatment of chronic hepatitis C: bonuses and challenges. Int J Med Sci.

